# Selective cytotoxicity of *Gomortega keule* essential oil through a ROS-mediated pro-apoptotic mechanism

**DOI:** 10.3389/fphar.2025.1722619

**Published:** 2025-12-08

**Authors:** Alejandro Madrid, Valentina Silva, Alessandra Russo, Alejandra Catalina Moller, Elizabeth Sánchez, Joan Villena, Carlos Jara-Gutiérrez, Iván Montenegro

**Affiliations:** 1 Laboratorio de Productos Naturales y Síntesis Orgánica (LPNSO), Facultad de Ciencias Naturales y Exactas, Universidad de Playa Ancha, Valparaíso, Chile; 2 Millennium Nucleus Bioproducts, Genomics and Environmental Microbiology (BioGEM), Valparaíso, Chile; 3 Department of Drug and Health Sciences, University of Catania, Catania, Italy; 4 Escuela de Tecnología Médica, Facultad de Medicina, Universidad de Valparaíso, Viña del Mar, Chile; 5 Centro de Biotecnología, Universidad Técnica Federico Santa María, Valparaiso, Chile; 6 Center of Interdisciplinary Biomedical and Engineering Research for Health (MEDING), Escuela de Medicina, Facultad de Medicina, Universidad de Valparaíso, Viña del Mar, Chile; 7 Center of Interdisciplinary Biomedical and Engineering Research for Health (MEDING), Escuela de Kinesiología, Facultad de Medicina, Universidad de Valparaíso, Valparaíso, Chile; 8 Center of Interdisciplinary Biomedical and Engineering Research for Health (MEDING), Escuela de Obstetricia y Puericultura, Facultad de Medicina, Universidad de Valparaíso, Viña del Mar, Chile

**Keywords:** apoptosis, cytotoxic activity, diterpenes, essential oil, *Gomortega keule*

## Abstract

Cancer is a leading cause of mortality worldwide, prioritizing the search for new therapies with improved toxicity profiles. Natural products, such as essential oils (EOs), are a valuable source of potential chemotherapeutic agents. *Gomortega keule*, a Chilean endemic tree, has traditional uses, but its cytotoxic potential remains unexplored. This study investigated the chemical composition and cytotoxic activity of *Gomortega keule* leaf EO. The chemical analysis revealed a unique profile rich in diterpenes (>50%), mainly phyllocladene (28.08%) and kaur-16-ene (19.74%), suggesting a distinct chemotype. The EO demonstrated potent cytotoxic activity against breast (MCF-7), prostate (PC-3), and colon (HT-29) cancer cell lines, with IC_50_ values of 3.97, 2.43, and 9.76 μg/mL, respectively. Remarkably, the EO exhibited exceptional selectivity, proving significantly more toxic to cancer cells than to non-tumorigenic cells. Specifically, it achieved a Selectivity Index (SI) of 24.01 for breast cancer cells compared to normal MCF-10A cells. Crucially, this selectivity profile significantly outperformed standard chemotherapeutic agents (daunorubicin and 5-fluorouracil), which displayed high toxicity towards healthy cells in this model. The mechanism of action involves the selective induction of reactive oxygen species (ROS), leading to mitochondrial membrane depolarization (ΔΨm) and caspase activation, culminating in apoptotic cell death. These findings highlight *G. keule* EO as a promising source for developing selective cytotoxic agents.

## 1 Introduction

Malignant neoplasms represent one of the main causes of morbidity and mortality worldwide. Projections from the GLOBOCAN project estimate that the global cancer burden reached nearly 20 million new cases in 2022, a figure expected to rise to 35 million by 2050 (Bray et al., 2024). Within this landscape, colorectal cancer ranks as the third most common cancer in the world (Ferlay et al., 2012; World Health Organization, 2023). Meanwhile, breast cancer is the most diagnosed neoplasm in women, and prostate cancer is the most commonly diagnosed in men, both representing a significant portion of the global oncological burden in terms of incidence and mortality (Bray et al., 2024; Palshof et al., 2024; Zahed et al., 2024). Conventional therapies, such as surgery, radiotherapy, and chemotherapy, remain the mainstay of treatment. However, their efficacy is often limited by the appearance of significant adverse side effects and the development of chemoresistance, a phenomenon that contributes to therapeutic failure in approximately 90% of patients with metastatic disease (Fernandez-Muñoz et al., 2025). This limitation has prompted the search for alternative and complementary therapies, with a particular focus on natural products that may act as chemotherapeutic or chemopreventive agents with more favorable toxicity profiles.

In this context, natural products of plant origin have re-emerged as an invaluable source of new bioactive compounds (Sharma et al., 2022). For colorectal cancer, the use of compounds such as curcumin and resveratrol as adjuvants to improve the response to standard chemotherapy has been extensively investigated (Fernandez-Muñoz et al., 2025). Furthermore, essential oils (EOs) have shown promise as a therapeutic tool, with studies demonstrating their cytotoxic, antiproliferative, and antimetastatic effects on cell lines of this cancer type (Garzoli et al., 2022). The use of oils like lavender has even been explored to improve the quality of life for patients with a colostomy (Duluklu and Şenol Çelik, 2019). Similarly, in breast cancer, EOs have been the subject of numerous systematic reviews confirming their pharmacological activity. Compounds such as monoterpenes and sesquiterpenes have been shown to induce apoptosis and inhibit cell proliferation (Mustapa et al., 2022). Specific studies, such as one conducted with *Oliveria decumbens* EO, have revealed not only pro-apoptotic effects but also a potent immunomodulatory effect, suggesting that its mechanism of action extends beyond direct cytotoxicity and may involve the activation of the host immune system (Jamali et al., 2020). Regarding prostate cancer, where the use of complementary and alternative medicines is particularly common among patients (Patterson et al., 2002), various natural products such as pomegranate extract, green tea, and curcumin have been studied (Klempner and Bubley, 2012). Recently, ethnopharmacological research has explored native South American plants; for example, the *Fabiana imbricata* EO, a Patagonian plant, has been shown to induce apoptosis in prostate cancer cells through the generation of reactive oxygen species (Madrid et al., 2025).

Ethnopharmacological knowledge, which explores the traditional uses of medicinal plants, is a fundamental tool for guiding the discovery of new therapeutic agents (Manosroi et al., 2006). A notable example is found in southern Chile: *G. keule* (Mol.) Baillon, an endemic tree commonly known as Queule. This species, the sole representative of the Gomortegaceae family, is considered a botanical relict and an ancient lineage. It is an evergreen tree that can reach up to 30 m in height, with a discontinuous distribution in the Coastal Range between the Maule and Biobío regions (Muñoz-Concha and Garrido-Werner, 2011; Crowley, 2020). This tree not only holds great scientific interest but also has deep cultural roots in the cosmovision of the Mapuche people, where it is considered a sacred tree, a source of strength and energy (newen) (Torri, 2010). Its traditional use in popular medicine, including the preparation of beverages from its yellow fruits, has been well documented (Muñoz-Concha and Garrido-Werner, 2011; Crowley, 2020). Modern science has begun to validate this ancestral knowledge; on one hand, it has been demonstrated that its EOs possess potent antioxidant activity (Simirgiotis et al., 2013). On the other hand, its antifungal efficacy has been confirmed against phytopathogenic fungi (Becerra et al., 2010) and, more directly relevant to human health, against yeasts of the genus *Candida* (Montenegro et al., 2025). However, its cytotoxic potential remains largely unexplored. Building upon its rich ethnopharmacological history and its already validated biological activities, the present study aims to investigate the potential of *Gomortega keule* EO as a cytotoxic agent against prostate, colon, and breast cancer. Specifically, this work evaluates the oil’s cytotoxic and selective activity, and delves into its pro-apoptotic mechanism to offer the first comprehensive assessment of its value as a source of novel cytotoxic compounds.

## 2 Materials and methods

### 2.1 Chemicals and reagents

Daunorubicin (CAS 23541-50-6), 5-fluorouracil (5-FU; CAS 51-21-8), and 2,2'-azobis(2-amidinopropane) dihydrochloride (AAPH; CAS 2997-92-4) were purchased from Sigma-Aldrich (St. Louis, MO, USA). Stock solutions of these agents were prepared in dimethyl sulfoxide (DMSO) or sterile water according to the manufacturer’s instructions. For biological assays, the *G. keule* EO was dissolved in ethanol (absolute grade, Merck, Darmstadt, Germany) to create stock solutions. It is important to note that while dichloromethane was used as the solvent for GC/MS analysis to ensure optimal volatility for chemical profiling, ethanol was selected for cell culture experiments due to its lower cytotoxicity and compatibility with live-cell assays. The final concentration of ethanol in the culture medium never exceeded 0.1% (v/v) to ensure no interference with cell viability.

### 2.2 Plant material

Leaves of *G. keule* were collected during the winter (July 2024) near the locality of Taiguén (32°36′44″S 71°03′51″W), Talcahuano, Biobío Region, Chile, at an altitude of approximately 70 m a.s.l. Botanical identification and authentication was verified by Mr. Patricio Novoa, and a voucher specimen (GQ-0724) was deposited at the Natural Products and Organic Synthesis Laboratory of Universidad de Playa Ancha, Valparaíso, Chile. Once in the laboratory, the fresh leaves were selected for their uniformity and absence of damage, washed with distilled water to remove surface residues, and dried with absorbent paper. Subsequently, the material was divided into two batches for the corresponding analyses.

### 2.3 Preparation of *Gomortega keule* EO

The *G. keule* EO was obtained from 500 g of fresh leaves by hydrodistillation for 5 h, using a Clevenger-type apparatus. (Madrid et al., 2025). The resulting hydrolate was then purified by liquid-liquid partition in a separator funnel with three successive 10 mL portions of ethyl acetate Finally, the purified EO was stored at 4 °C pending further chemical and biological analysis.

### 2.4 Chromatographic analysis of volatile compounds

Two complementary analyses were performed to characterize the volatile profile: one on fresh leaves to capture the most volatile compounds and another on the extracted EO to determine its majority composition.

#### 2.4.1 Analysis of fresh leaf volatiles by HS-SPME-GC/MS

2.5 g of fresh leaf fragments were placed in a headspace vial and equilibrated at 50 °C for 30 min. Subsequently, a solid-phase microextraction (SPME) fiber (50/30 µm DVB/Car-PDMS) was exposed to the headspace for 30 min to capture the volatile compounds. The fiber was desorbed in the gas chromatograph injector in splitless mode at 250 °C for 5 min.

#### 2.4.2 EO analysis by GC/MS

The *G. keule* EO was diluted to 1% (v/v) in dichloromethane and homogenized by vortexing. Subsequently, 1 µL of the sample was injected for chromatographic analysis.

#### 2.4.3 Chromatographic conditions, compound identification, and quantification

Both analyses were performed on a Thermo Scientific Trace 1310 gas chromatograph coupled to an ISQ, LT mass spectrometer. Chromatographic separation was carried out on a Restek RTX-5MS column (30 m × 0.25 mm ID × 0.25 µm), using helium as the carrier gas at a constant flow rate of 1.0 mL/min. The oven temperature program was as follows: initial temperature of 40 °C for 1 min, followed by a ramp to 200 °C and held for 5 min, and a second ramp to 250 °C at 8 °C/min, holding for 5 min. The mass spectrometer operated in electron impact (EI) mode at 70 eV, performing a full scan over a mass range of 50–450 amu. The identification of volatile compounds was performed by comparing the obtained mass spectra with the NIST 2021 library, considering positive identifications for compounds with a Similarity Index (SI) greater than 800. Additionally, identities were confirmed by comparing the calculated Kovat Indices with those reported in the literature. Finally, the relative percentage composition of the EO components was calculated from the chromatographic peak areas using the area normalization method.

### 2.5 Cytotoxicity activity

#### 2.5.1 Cells

Human cancer cell lines, MCF-7 (human mammary gland adenocarcinoma), HT-29 (human colorectal adenocarcinoma), PC-3 (human prostate adenocarcinoma) and normal human cell lines; CCD 841 CoN (colon epithelial), and MCF-10A (epithelial mammary gland) were obtained from American Type Culture Collection (Rockville, MD, USA). All tested cell lines were maintained in a 1:1 mixture of Dulbecco’s modified Eagle’s medium (DMEM) and Ham’s F12 medium, containing 10% heat-inactivated fetal bovine serum (FBS), penicillin (100 U/mL), and streptomycin (100 μg/mL) in a humidified atmosphere with 5% CO_2_ at 37 °C. The cells were plated at a constant density to obtain identical experimental conditions in the different tests, thus to achieve a high accuracy of the measurements.

#### 2.5.2 SRB bioassay

Sulforhodamine B (SRB) assay was performed as already described (Silva et al., 2025). To assess cell viability, cells were seeded at 3 × 10^3^ cells per well in 96-well plates and incubated at 37 °C with 5% CO_2_. The cells were treated with the EO at concentrations ranging from 1.25 to 100 μg/mL for 72 h. Following treatment, cells were fixed with 10% trichloroacetic acid, stained with 0.1% SRB, and then washed to remove unbound stain. The protein-bound stain was solubilized, and cell density was determined by measuring fluorescence at 540 nm. Daunorubicin and 5-fluorouracil were used as positive controls. Values shown are the mean ± standard deviation of three independent experiments performed in triplicate.

#### 2.5.3 Selectivity index

The selectivity index (SI) was determined by the ratio between the IC_50_ value of the cytotoxicity obtained for normal cells and the value found for a selected cancer cell line, as shown in Equation 1:

$$SI=\text{IC}50\;\left(\text{Normal Cell}\right)/\text{IC}50\;\left(\text{Cancer Cell}\right)$$ (1)

Where a SI > 3 was considered to belong to a selective sample (Moller et al., 2021).

#### 2.5.4 Intracellular ROS generation

Intracellular ROS production was assessed by flow cytometry. Cells were seeded in 24-well plates at a density of 16 × 10^4^ cells/well in 500 µL of culture medium. The determination of ROS was performed following the methodology of Villena et al. (2021) using a fluorescent probe 2′,7′-dichlorofluorescein diacetate (DCFH-DA). Briefly, cells were treated with the *G. keule* EO at concentrations of 5, 10, and 20 μg/mL for a total of 24 h. After treatment, cells were further incubated with 10 μM DCFH-DA at 37 °C for 30 min. Subsequently, cells were harvested, rinsed, re-suspended in PBS and analyzed for 2′,7′-dichlorofluorescein (DCF) fluorescence by flow cytometry (FacScalibur, Beckton Dickinson).

#### 2.5.5 Analysis of mitochondrial membrane potential (ΔΨm)

Changes in mitochondrial membrane potential (ΔΨm) were measured using the cationic fluorescent probe Rhodamine 123, as previously described (Villena et al., 2021). Briefly, exponentially growing cells were treated with *G. keule* EO as indicated in the figure legends. During the final 60 min of incubation, cells were labeled with 1 µM Rhodamine 123. After treatment, cells were washed with ice-cold PBS, detached by trypsinization, and immediately analyzed by flow cytometry. The results are expressed as the percentage of cells retaining Rhodamine 123 fluorescence, which corresponds to the cell population with intact mitochondrial membrane potential.

#### 2.5.6 Measurement of caspase activity

Caspase activity, an indicator of apoptosis, was measured using the CaspACE™ FITC-VAD-FMK *in situ* marker (Promega, Santiago, Chile), as described previously (Jara-Gutiérrez et al., 2024). Cells were treated with the *G. keule* EO (5 and 10 μg/mL) for 48 h. During the final 20 min of the treatment, cells were incubated with the FITC-VAD-FMK reagent in darkness at room temperature. Following incubation, the cells were washed twice with PBS, harvested by trypsinization, and pelleted by centrifugation (1500×g for 10 min). The resulting cell pellet was then resuspended in fresh PBS for immediate analysis by flow cytometry, with fluorescence detected using the FL3 filter. Results are expressed as the percentage of FITC-positive cells, representing the cell population undergoing apoptosis.

### 2.6 Statistical analysis

All *in vitro* assays were performed in three independent biological replicates, and each replicate was carried out in triplicate. The results are expressed as mean values ± Standard Deviation (SD). Statistical significance was defined as *p* < 0.05. Following the protocol described by Jara-Gutiérrez et al. (2024), and due to the non-parametric nature of the data, the results were analyzed using a Kruskal-Wallis ANOVA with a confidence level of 95% using STATISTICA 7.0 software.

## 3 Results and discussion

### 3.1 Profile of volatile compounds from fresh leaves (HS-SPME)

**FIGURE 1:**
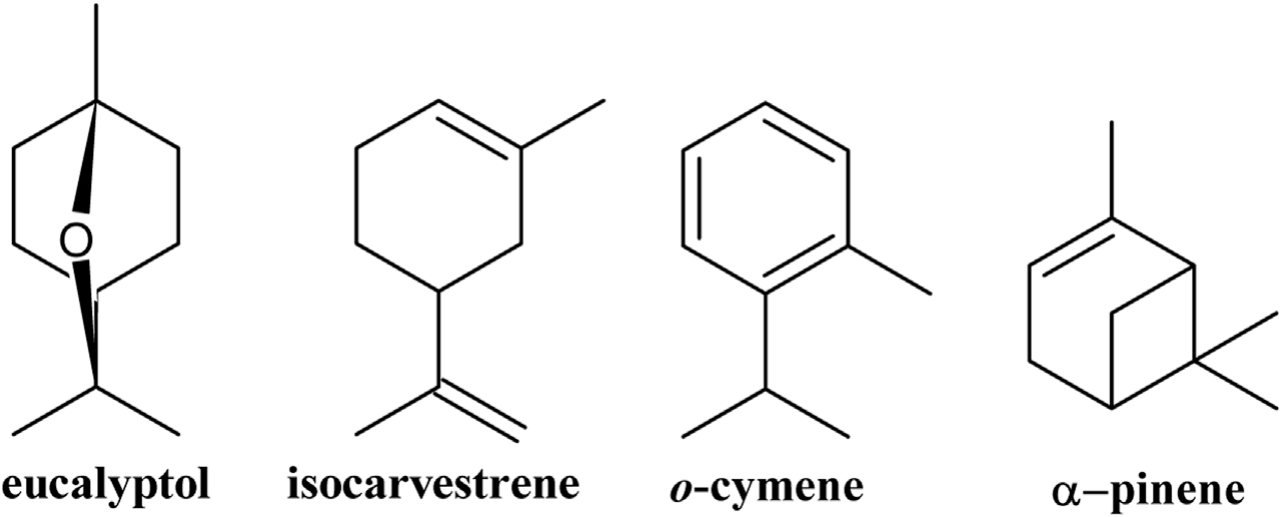
Major volatile compounds present in the leaves of *Gomortega keule*.

The HS-SPME analysis of fresh *G. keule* leaves identified a total of 10 compounds, which corresponded exclusively to hydrocarbon (60.84%) and oxygenated (35.71%) monoterpenes. Of these, the major components were eucalyptol (33.68%), *o*-cymene (25.28%), isocarvestrene (12.10%), and alpha-pinene (10.14%), as detailed in Table 1 and Figure 1.

**TABLE 1:** SPME profile of the *Gomortega keule* leaves.

N°	RT (min)	Components	% Area^a^	RI^a^	RL^b^	Identification
1	9.09	α-pinene	10.14	930	930	RL, MS, Co
2	9.62	Camphene	0.50	941	941	RL, MS, Co
3	10.54	Sabinene	4.68	956	956	RL, MS, Co
4	10.64	β-pinene	7.18	970	970	RL, MS, Co
5	12.37	*o*-cymene	25.28	1014	1014	RL, MS, Co
6	12.50	Isocarvestrene	12.10	1026	1027	RL, MS
7	12.61	Eucalyptol	33.68	1033	1033	RL, MS, Co
8	14.58	Dehydro-*p*-cymene	0.96	1070	1070	RL, MS, Co
9	16.20	Pinocarveol	0.27	1133	1134	RL, MS
10	17.40	4-terpinenol	1.76	1160	1160	RL, MS, Co
		Total identified	96.55			
		Oxygenated monoterpenes	35.71			
		Hydrocarbon monoterpenes	60.84			

^a^ Experimental retention index for non-polar column.

^b^ Bibliographic retention index for non-polar column, MS, mass spectra.

This is the first time that volatiles emitted from the fresh leaves of *G. keule* have been reported, which contributes to the phytochemical knowledge of this ancestral tree.

### 3.2 Composition of *Gomortega keule* EO

The *G. keule* EO, extracted from its fresh leaves, was obtained with a yield of 1.22% (v/w) and is mainly composed of hydrocarbon diterpenes (54,77%), followed by hydrocarbon sesquiterpenes (18.81%), and oxygenated sesquiterpenes (9.95%) (Table 2).

**TABLE 2:** EO composition of *Gomortega keule*.

N°	RT (min)	Components	% Area^a^	RI^a^	RL^b^	Identification
1	7.54	Viridiflorene	1.24	1492	1493	RL, MS
2	7.81	Not identified	0.07			
3	7.95	Calamenene	2.85	1516	1517	RL, MS
4	8.34	α-calacorene	7.69	1548	1548	RL, MS
5	8.53	Diepicedrene-1-oxide	0.33	1551	1551	RL, MS
6	8.61	Not identified	0.61			
7	8.68	Not identified	0.89			
8	9.00	(−)-Spathulenol	1.01	1574	1574	RL, MS, Co
9	9.14	Globulol	2.30	1580	1580	RL, MS
10	9.30	Cubeban-11-ol	0.99	1587	1588	RL, MS
11	9.33	Not identified	0.98			
12	9.57	Not identified	0.28			
13	9.78	α-corocalene	3.59	1605	1605	RL, MS
14	9.92	Epicubenol	0.86	1626	1627	RL, MS
15	10.17	Not identified	0.77			
16	10.21	Not identified	0.43			
17	10.47	Not identified	0.30			
18	10.49	Not identified	0.39			
19	10.75	Not identified	0.24			
20	10.92	Cadalene	14.92	1643	1643	RL, MS
21	11.21	Not identified	0.13			
22	11.58	Not identified	0.82			
23	11.61	Not identified	0.79			
24	12.25	Not identified	0.16			
25	12.32	Not identified	0.25			
26	12.59	Not identified	0.60			
27	12.61	Not identified	0.36			
28	13.01	Not identified	0.24			
29	13.69	Not identified	0.44			
30	13.72	Not identified	0.18			
31	16.29	Rimuene	2.39	1884	1885	RL, MS
32	17.84	Pimaradiene	4.56	1930	1931	RL, MS
33	19.58	Phyllocladene	28.08	2012	2012	RL, MS
34	20.18	Kaur-16-ene	19.74	2040	2040	RL, MS
35	33.55	Not identified	0.42			
		Total identified	90.55			
		Total not identified	9.45			
		Hydrocarbon sesquiterpenes	30.29			
		Oxygenated sesquiterpenes	5.49			
		Hydrocarbon diterpenes	54.77			

^a^ Experimental retention index for non-polar column.

^b^ Bibliographic retention index for non-polar column, MS, mass spectra.

**FIGURE 2:**
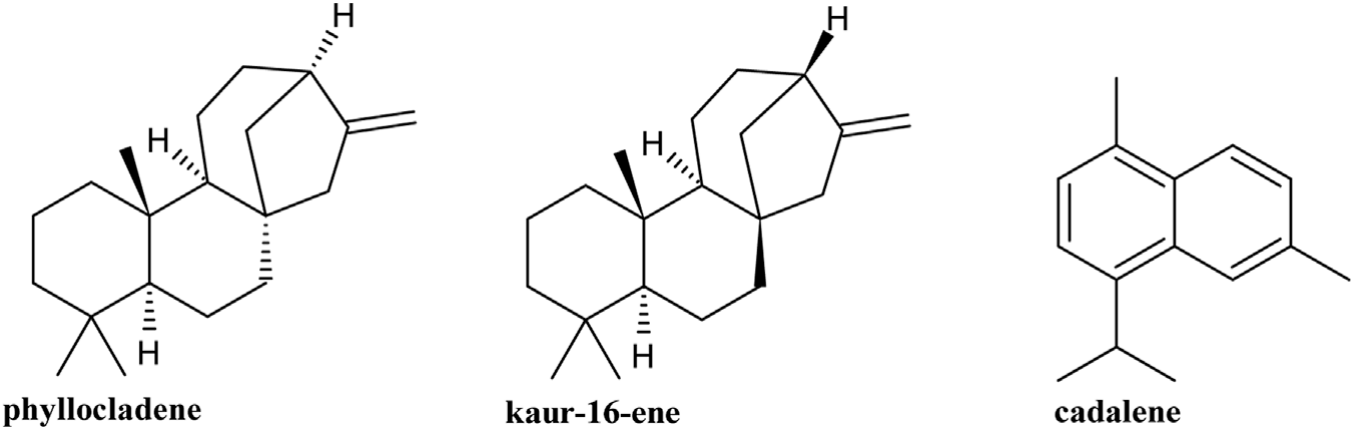
Major compounds present in *Gomortega keule* EO.

Fourteen compounds were identified in the *G. keule* EO, which corresponded to 90.55% of the total oil analyzed, and the main components were phyllocladene (28.08%), kaur-16-ene (19.74%), and cadalene (14.92%) (Figure 2).

The phytochemical analysis of *G. keule* leaves reveals a fascinating duality in its volatile compound profile, which is directly dependent on the analytical method used. The comparison between the profile obtained from fresh leaves by HS-SPME and the composition of the EO extracted by hydrodistillation shows drastic differences. Far from being contradictory, these results are complementary and offer a comprehensive view of the plant’s phytochemistry. HS-SPME analysis is a non-invasive and gentle technique that captures compounds emitted into the headspace at moderate temperatures. By its nature, this method is inherently biased towards more volatile, low-molecular-weight molecules (Souza-Silva, et al., 2015). Consequently, it is not surprising that the profile of the fresh leaves is exclusively dominated by monoterpenes (96.55%). This result represents the profile of volatiles that the leaf naturally emits into its environment, which could be related to ecological functions such as defense against herbivores or chemical communication. On the other hand, hydrodistillation is an exhaustive extractive method that uses high temperatures (100 °C) and steam entrainment over a prolonged period. This process forces the release of less volatile, higher-molecular-weight compounds stored in the plant’s glandular structures, such as sesquiterpenes and, notably, diterpenes (Siddiqui et al., 2024). The complete absence of the monoterpenes detected by SPME in the EO is a key finding, likely because these compounds, due to their extreme volatility and higher water solubility, were lost through evaporation or remained dissolved in the aqueous phase during the energetic distillation process (Masango, 2005). Thus, the two phytochemical profiles are not mutually exclusive: the HS-SPME analysis reveals the natural “aroma” of the fresh leaf, while the EO analysis shows the total content of stored semi-volatile and heavy metabolites. This distinction is fundamental. This study reveals significant differences in both the yield and the phytochemical profile of *G. keule* EO compared to previously published data, suggesting the existence of distinct chemotypes. Specifically, the obtained yield was 1.21% (v/w), an intermediate value between those reported by Becerra et al. (2010) (1.43%) and Montenegro et al. (2025) (0.99%). These variations in yield are expected and can be attributed to environmental and geographical factors that influence the production of secondary metabolites (Moghaddam et al., 2023; Dobhal et al., 2024; Jojić et al., 2024). However, the most notable difference lies in the chemical composition. Our EO is distinguished by a unique profile with a predominance of diterpenes (>50%), a very uncommon characteristic in EOs due to the high molecular weight and low volatility of these compounds (Wani et al., 2021). This profile contrasts sharply with the findings of Montenegro et al. (2025), whose oil was dominated by oxygenated monoterpenes (>60%), such as eucalyptol and 4-terpineol, with a minimal presence of diterpenes (<5%). Despite these differences, a point of structural connection exists: the study by Becerra et al. (2010) reported 20% kaurene, a diterpene that shares the same base skeleton as the kaur-16-ene identified in both our study and that of Montenegro et al. (2025). This combination of radically different phytochemical profiles with comparable extraction yields is the most compelling evidence for the existence of genetically determined chemotypes in *G. keule* (Martinelli et al., 2023).

### 3.3 Cytotoxic activity

The *G. keule* EO exhibited potent cytotoxic activity against all cancer cell lines tested, with the corresponding IC_50_ values presented in Table 3.

**TABLE 3:** Cytotoxicity (IC_50_) and selectivity index (SI) of *Gomortega keule* EO compared to standard drugs.

Sample	Cell line	IC_50_ (µg/mL)	SI
*G. Keule* EO	MCF-7	3.97 ± 0.67	24.01^a^
	PC-3	2.43 ± 0.73	20.30^b^
	HT-29	9.76 ± 1.03	5.06^b^
	CCD 841 CoN	49.34 ± 0.33	—
	MCF-10A	95.32 ± 0.54	—
Daunorubicin	MCF-7	1.86 ± 0.05	1.15^a^
	PC-3	1.32 ± 0.04	9.14^b^
	HT-29	19.32 ± 0.50	0.62^b^
	CCD 841 CoN	12.07 ± 0.40	—
	MCF-10A	2.13 ± 0.41	—
5-FU	MCF-7	22.30 ± 0.20	0.15^a^
	PC-3	16.42 ± 0.60	2.54^b^
	HT-29	8.90 ± 0.70	4.69^b^
	CCD 841 CoN	41.71 ± 0.30	—
	MCF-10A	3.25 ± 0.23	—

Results are expressed as mean ± standard deviation (SD) of three independent experiments performed in triplicate (n = 3). IC_50_: Concentration required to inhibit cell proliferation by 50%. SI (Selectivity Index) = IC_50_ Normal Cell/IC_50_ Cancer Cell.

^a^ SI, calculated using MCF-10A, as the specific normal breast reference line.

^b^ SI, calculated using CCD, 841 CoN as the reference line.

The *G. keule* EO exhibited potent cytotoxic activity against breast (MCF-7), prostate (PC-3), and colon (HT-29) cancer cell lines, with IC_50_ values of 3.97 ± 0.67, 2.43 ± 0.73, and 9.76 ± 1.03 μg/mL, respectively. All of these values are well below the 30 μg/mL threshold established by the U.S. National Cancer Institute (NCI) to consider an extract a promising candidate with cytotoxic potential (Canga et al., 2022). However, while potent cytotoxicity is a crucial first step, the true therapeutic potential of a compound lies in its ability to selectively target cancer cells while sparing healthy ones. This critical aspect is measured by the selectivity index (SI), where a value greater than 3 is considered indicative of meaningful selectivity (Mahmood et al., 2024).

To investigate this, we performed a tissue-specific comparison for breast cancer, evaluating the EO’s effect on the MCF-7 cancer line against its non-tumorigenic counterpart, the MCF-10A line. The outcome was remarkable, revealing an exceptional SI of 24.01, which confirms that the EO is profoundly more toxic to breast adenocarcinoma cells than to healthy epithelial cells from the same tissue. This favorable selectivity profile was not limited to breast cancer. The EO also showed a strong selective action against colon cancer (SI = 5.06) when comparing the HT-29 and CCD 841 CoN lines. For the PC-3 prostate cancer line, the CCD 841 CoN line served as the healthy control in the absence of a non-tumorigenic prostate line in our panel. Despite being a cross-tissue comparison, this test yielded a remarkably high SI of 20.30, further reinforcing the EO’s promising safety profile and its specific action against malignant cells.

To properly contextualize these findings, the EO’s performance was benchmarked against standard chemotherapeutic agents (Table 3). The *G. keule* EO exhibited an exceptional SI of 24.01 for breast cancer cells when compared to the tissue-specific normal line MCF-10A. In sharp contrast, the standard drugs showed significantly lower selectivity in this specific model. Daunorubicin presented an SI of 1.15, while 5-FU showed an SI of 0.15 against the MCF-10A line, due to their high toxicity towards healthy breast epithelial cells (IC_50_ values of 2.13 and 3.25 μg/mL, respectively). This remarkable difference highlights the *G. keule* EO as a promising candidate with a potentially superior safety profile and a wider therapeutic window compared to these conventional agents in this *in vitro* model.

It is important to acknowledge a specific limitation in our selectivity analysis regarding prostate cancer. The Selectivity Index (SI) for the PC-3 cell line was calculated using the non-tumorigenic colon epithelial line (CCD 841 CoN) as a reference, due to the unavailability of a specific normal prostate cell line in our panel. While this comparison provides a valid estimate of the oil’s general toxicity towards healthy epithelial cells, it constitutes a cross-tissue comparison. Therefore, the reported selectivity for prostate cancer should be interpreted with caution, as a tissue-specific model (e.g., using RWPE-1 or PNT cell lines) would provide a more precise assessment of the therapeutic window for this specific cancer type (Bello et al., 1997; Rumsby et al., 2011).

**FIGURE 3:**
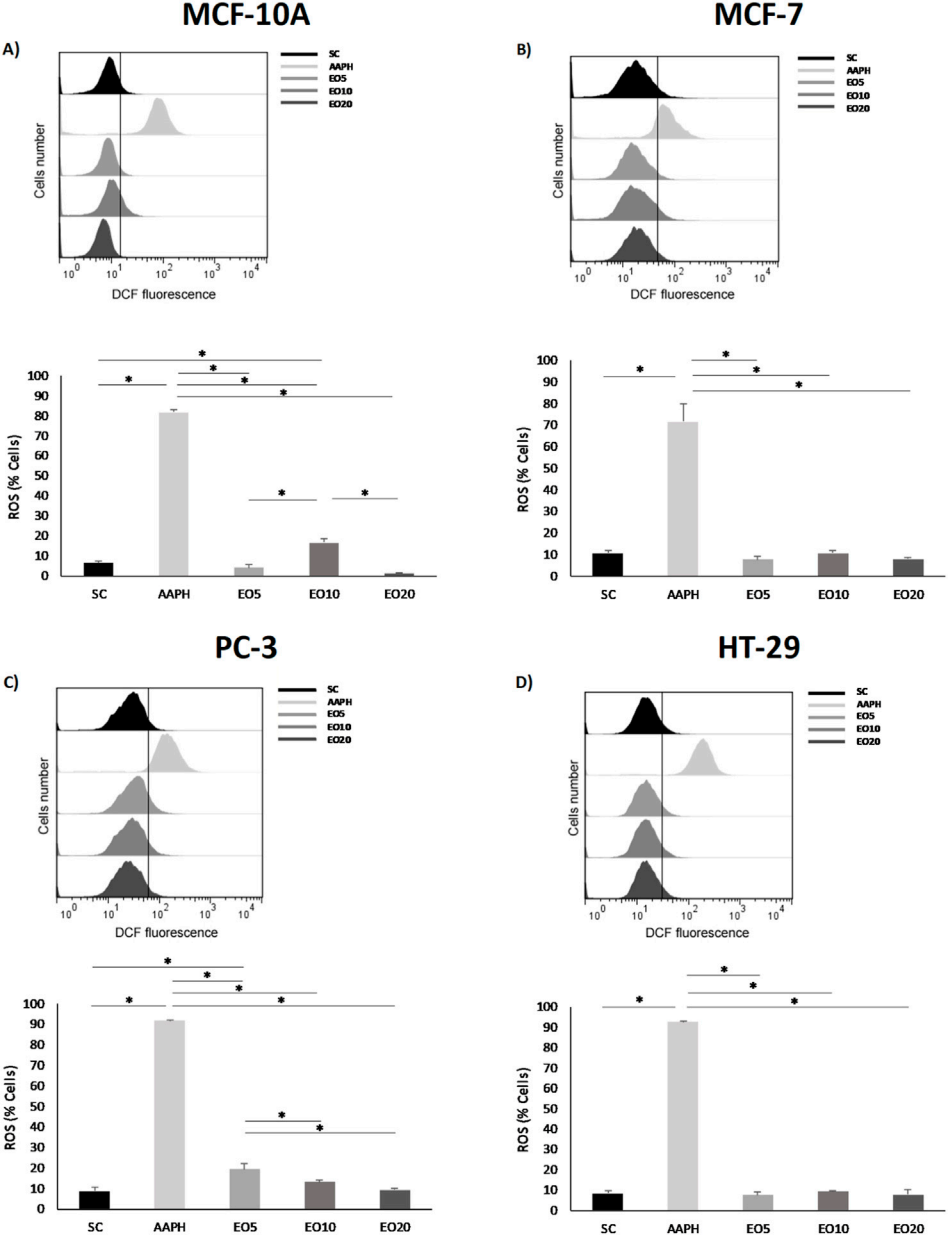
Reactive oxygen species (ROS) production was measured in nontumor breast cell line [**(A)** = MCF-10A] and cancer cell lines [**(B)** = MCF-7, **(C)** = PC-3 and **(D)** = HT-29] after being exposed for 24 h to three different concentrations of *Gomortega keule* EO. Treatments: EO5 = 5 μg/mL, EO10 = 10 μg/mL, and EO20 = 20 μg/mL. For ROS production control, 10 µM AAPH (AAPH) was used as a positive control and 0.1% ethanol as a solvent control (SC). **(A–D)** Show the mean percentage of cells with ROS production. Data represent the mean ± standard deviation (SD) of three independent experiments performed in triplicate (n = 3). Statistical significance was determined by Kruskal-Wallis ANOVA. *p < 0.05 compared to the solvent control (SC).

Building on these compelling activity and selectivity results, we sought to understand the underlying mechanism of action. A central finding is that the cytotoxic activity of the *G. keule* EO is directly associated with the induction of intracellular oxidative stress. To evaluate this, we utilized AAPH (2,2'-azobis(2-amidinopropane) dihydrochloride) as a positive control, given its well-established role as a peroxyl radical generator that mimics oxidative stress conditions. As shown in Figure 3, the treatment with *G. keule* EO induced a differential response in intracellular ROS levels. A significant and concentration-dependent increase was clearly observed in PC-3 cells (Figure 3C) compared to the solvent control (CS). In contrast, in MCF-7 and HT-29 cells (Figures 3B,D), ROS levels measured at 24 h remained comparable to the solvent control. This suggests that while oxidative stress appears to be a primary driver of cytotoxicity in prostate cancer cells, the mechanism in breast and colon cancer cells may involve different kinetic profiles or alternative upstream triggers for the observed mitochondrial depolarization. To validate this, the MCF-10A cell line was strategically chosen for comparative flow cytometry studies precisely because of its high resistance (high IC_50_ value). This resilience made it the ideal negative control, allowing us to analyze cellular mechanisms like ROS production at EO concentrations that are lethal to cancer cells. This finding, which confirms that the pro-oxidant effect is a specific mechanism, aligns with a growing body of literature on the action of EOs. For instance, Kong et al. (2022) also documented that various EOs can alter the cellular redox balance to induce biological damage in target cells.

**FIGURE 4:**
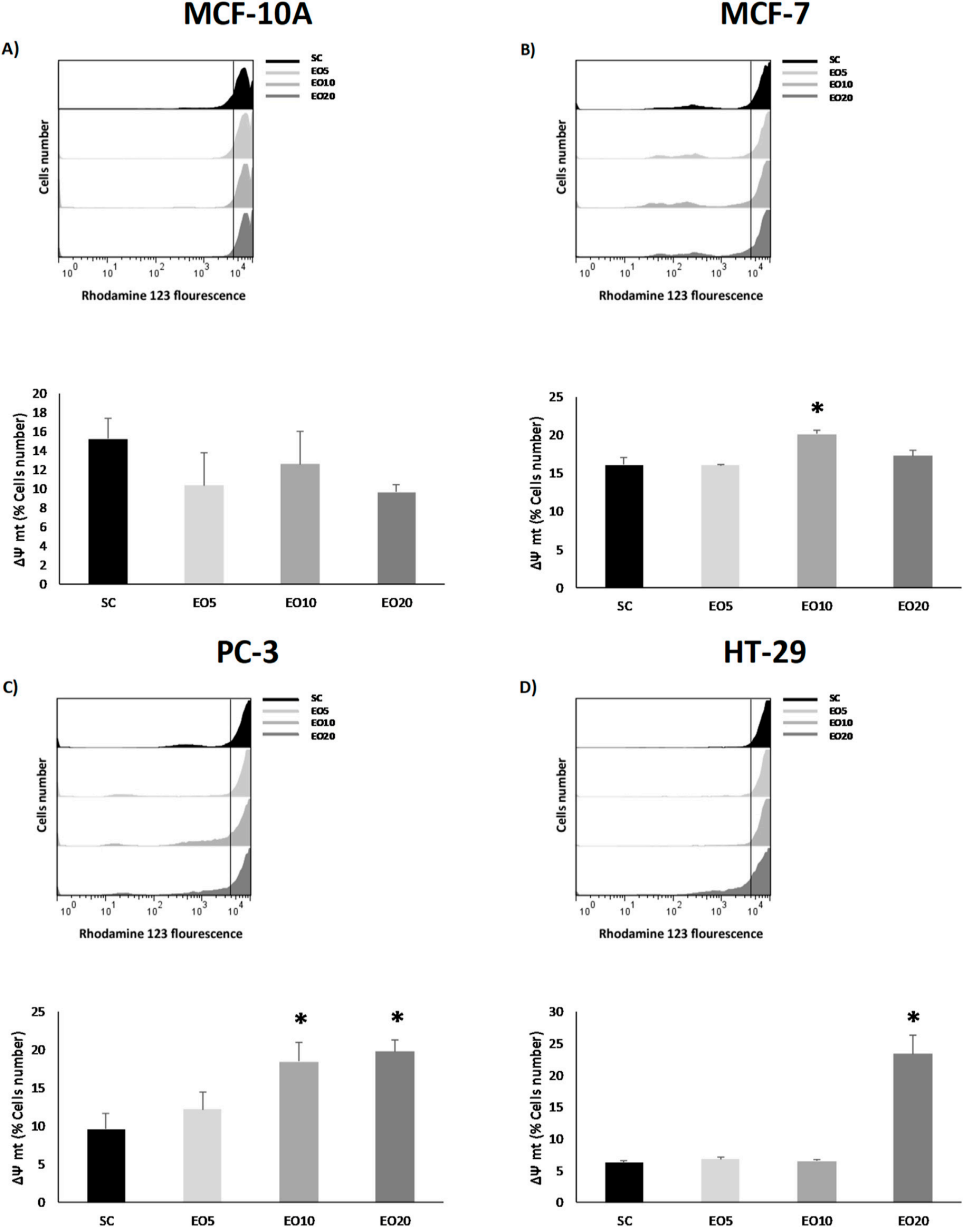
Mitochondrial membrane potential (ΔΨm) was analyzed in nontumor [**(A)** = MCF-10A] and cancer cell lines [**(B)** = MCF-7, **(C)** = PC-3 and **(D)** = HT-29] after being exposed for 48 h to three different concentrations of *Gomortega keule* EO. Treatments: EO5 = 5 μg/mL, EO10 = 10 μg/mL, and EO20 = 20 μg/mL 0.1% ethanol was used as solvent control (SC). **(A–D)** show the mean percentage of cells retaining ΔΨm. Data represent the mean ± standard deviation (SD) of three independent experiments performed in triplicate (n = 3). Statistical significance was determined by Kruskal-Wallis ANOVA. *p < 0.05 compared to the solvent control (SC).

This oxidative stress often leads to mitochondrial dysfunction, a key event in apoptosis. In accordance with this, our results demonstrate a selective loss of mitochondrial membrane potential (ΔΨm) only in *the* cancer cell lines (MCF-7, PC-3, and HT-29), as evidenced in Figure 4. This finding aligns perfectly with recent studies, such as that on *Cinnamomum zeylanicum* oil, which was also shown to inhibit melanoma cell proliferation by increasing ROS and causing mitochondrial membrane depolarization selectively in cancer cells, but not in normal human cells (PBMCs) (Cappelli et al., 2023). The loss of ΔΨm is a point-of-no-return in the intrinsic apoptosis pathway, as it leads to the release of pro-apoptotic factors from the mitochondria into the cytosol (Blowman et al., 2018).

**FIGURE 5:**
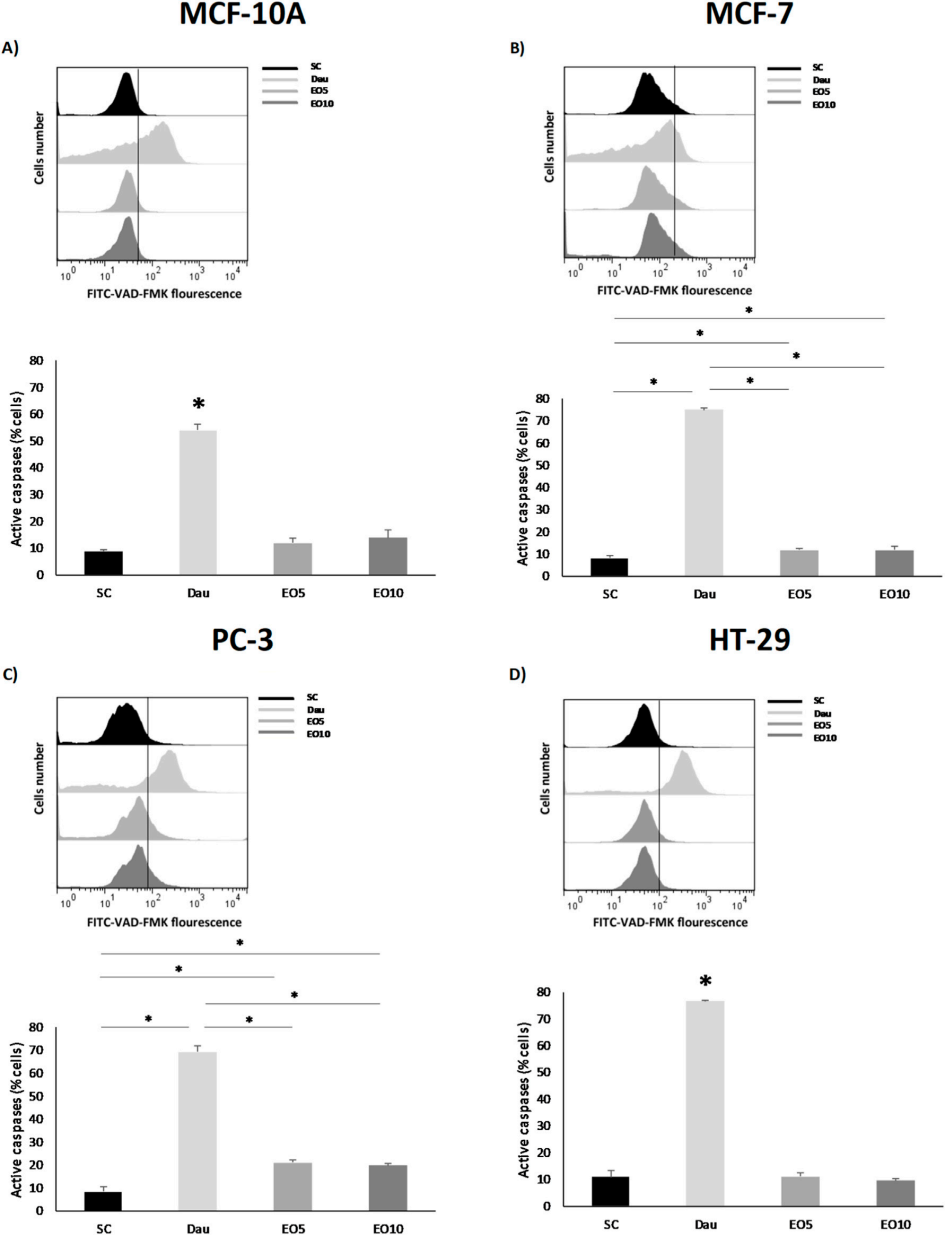
Caspase activity was analyzed in nontumor [**(A)** = MCF-10A] and cancer cell lines [**(B)** = MCF-7, **(C)** = PC-3 and **(D)** = HT-29] after being exposed for 48 h to two different concentrations of *Gomortega keule* EO. Treatments: EO5 = 5 μg/mL and EO10 = 10 μg/mL. For caspase activation control, 1 µM daunorubicin (Dau) was used as a positive control and 0.1% ethanol as solvent control (SC). **(A–D)** Show the mean percentage of cells with active caspases. Data represent the mean ± standard deviation (SD) of three independent experiments performed in triplicate (n = 3). Statistical significance was determined by Kruskal-Wallis ANOVA. *p < 0.05 compared to the solvent control (SC).

This release of mitochondrial factors activates the caspase cascade, which involves the executioner proteases of apoptosis. Indeed, our analysis confirmed the activation of caspases in the breast (MCF-7) and prostate (PC-3) cancer cell lines following treatment with EO (Figure 5).

This result is consistent with studies on other EOs, such as that of *Origanum majorana*, which also induces apoptosis in lung and epidermoid carcinoma cells through the activation of caspase-3/7 (Gökhan, 2022). It is interesting to note that, although the colon (HT-29) cell line also showed a loss of ΔΨm, significant caspase activation was not detected under the same conditions. This could suggest that, in this cell line, the oil induces a different type of cell death (such as necrosis or a caspase-independent apoptotic pathway) or that caspase activation occurs at a later time point than the one analyzed. Taken together, these results delineate a clear mechanism of action for *G. keule* EO: the induction of ROS leads to mitochondrial damage (loss of ΔΨm), which in turn triggers caspase activation and apoptotic cell death, at least in breast and prostate cancer cells. This apoptotic pathway is a hallmark of the cytotoxic properties reported for many EOs and their terpenoid components. For instance, frankincense extracts have also been shown to induce cancer cell-specific cytotoxicity by activating caspases and inducing PARP cleavage (Blowman et al., 2018; Kong et al., 2022). Crucially, the selectivity of these effects towards tumor cells further reinforces the therapeutic potential of this EO as a source of natural cytotoxic compounds. Finally, it is important to acknowledge that while the high abundance of diterpenes suggests they play a major role in the observed cytotoxicity, the *G. keule* EO is a complex mixture. High diterpene contents, although uncommon due to their low volatility (Ben Miri, 2025), have been reported in other species such as *Euphorbia mauritanica*, which also shares the presence of kaur-16-ene (Essa et al., 2021). Current literature suggests that the biological activity of EOs often results from the synergistic interaction of their major and minor constituents, producing a combined effect greater than the sum of individual compounds (Essa et al., 2021; Ben Miri, 2025). Therefore, the potent and selective activity reported here is attributed to the *G. keule* EO as a whole.

## 4 Conclusion

The present study characterizes a unique chemotype of *Gomortega keule* EO, distinguished by a high prevalence of diterpenes such as phyllocladene and kaur-16-ene, which differs notably from previous reports. This EO demonstrated potent cytotoxic activity against breast, prostate, and colon cancer cell lines. While the dominant diterpenes likely contribute to this effect, the observed activity is attributed to the EO as a complex mixture, suggesting potential synergistic interactions among its constituents. Mechanistically, the cytotoxic effect is driven by the selective induction of oxidative stress (ROS) in tumor cells, leading to mitochondrial membrane depolarization and subsequent caspase-dependent apoptosis. Crucially, our comparative analysis revealed that the *G. keule* EO possesses exceptional selectivity towards breast cancer cells (SI > 24) when compared to normal mammary epithelial cells (MCF-10A). This selectivity profile significantly outperforms that of standard chemotherapeutic agents like daunorubicin and 5-fluorouracil in this specific model. These findings validate the ethnopharmacological relevance of *G. keule* and highlight its potential as a promising source of bioactive compounds with selective cytotoxic properties.

## Data Availability

The raw data supporting the conclusions of this article will be made available by the authors, without undue reservation.
